# Family-centeredness of services for young children with Down syndrome: an observational study from Turkey

**DOI:** 10.3906/sag-2009-76

**Published:** 2021-02-26

**Authors:** Emine Bahar BİNGÖLER PEKCİCİ, Ezgi ÖZALP AKIN, Funda AKPINAR, Gamze HAYRAN, Cansu KELEŞ, Betül YAĞBASAN, Nazmiye KURŞUN, İlgi ÖZTÜRK ERTEM

**Affiliations:** 1 Developmental-Behavioral Pediatrics Division, Department of Pediatrics, Faculty of Medicine, Ankara University, Ankara Turkey; 2 Department of Biostatistics, Faculty of Medicine, Ankara University, Ankara Turkey

**Keywords:** Down syndrome, disability, children with special needs, family-centered care, measure of process of care (MPOC)

## Abstract

**Background/aim:**

Physicians require information on the family centeredness of services for children with Down syndrome, one of the most frequently encountered disabilities in childhood. We aimed to determine the family-centeredness of services for young children with Down syndrome and using a bioecological theory framework we hypothesized that child, family and service-related factors would be associated with such services.

**Materials and methods:**

In a crosssectional design, children with Down syndrome seen at Ankara University Developmental Pediatrics Division (AUDPD) between February 2020 and June 2020 were included if they had received services in the community for at least 12 months. Mothers responded to the measure of process of care-20 (MPOC-20) used to measure family centeredness.

**Results:**

All 65 eligible children were included; 57% were boys and median age was 25.0 (IQR: 18.5–38.0) months. The MPOC-20 subscale scores were highest for the “respectful and supportive care (RSC)” (median 6.0; IQR: 4.8–6.8) and lowest for the “providing specific information” (median 3.0; IQR: 4.4–6.5) subscales. On univariate analyses, maternal education <high school was associated with scores ≤4 on the RSC (OR = 6.75; 95%CI = 1.77–25.64) and “enabling and partnership” subscales (OR = 3.10; 95%CI = 1.06–9.05); income ≤ minimum wage (OR=3.94; 95%CI=1.10-14.02) was associated with scores ≤4 on the RSC. In the multivariate logistic regression model, maternal education ≤ high school was independently associated with RSC scores ≤4 (OR = 5.13; 95%CI = 1.26–20.84).

**Conclusion:**

Our findings imply that limitations in family-centeredness of community service for young children with Down syndrome. Deficiencies of services particularly for children with less educated mothers need to be urgently resolved.

## 1. Introduction 

Down syndrome (DS) is one of the most common and well-recognized causes of intellectual disability, and family-centered care (FCC) is known to be the gold standard of service provision for children with DS and their families [1]. Physicians providing healthcare for children with DS are recommended to conduct ongoing assessments of whether they are receiving services that are family-centered [2]. Despite these recommendations and the commonness of this well-recognized disorder leading to disability, there is a dearth of research on family-centeredness of services for children with DS, particularly in low and middle-income countries. 

Family-centered care is a framework of values, attitudes, and approaches to services which recognizes that families are the experts on their child and that they must be seen as active partners in the management of their child’s difficulties and the provision of services [1,3]. For decades, family-centeredness has been the cornerstone of service delivery for children with developmental difficulties in pediatric health care [1,2]. A systematic review of 24 studies, all from high-income countries, has documented the impact of FCC on children with special health care needs and their families [4]. Services that provided FCC were associated with improved developmental outcomes and adjustment for the children, better family functioning, parental well‐being, parental perceptions of competency and satisfaction, and more efficient use of services. It was shown that the FCC improved children’s quality of life independent of disease severity [5]. In a review of 55 studies on FCC from 10 high-income countries, in general, parents of children with disabilities reported that service providers were respectful and provided comprehensive services in partnership with families [6]. It has been reported that the family-centeredness of care for children with disabilities may vary depending on the cultural context [7]. There have been three studies on FCC of children with disabilities from the Asian context. In the studies from Japan [8], Korea [9] and China [10] parents of children with disabilities attending rehabilitation centers reported that service providers were providing respectful and supportive care for their children. A study from Singapore reported that service providers that who had higher self-efficacy in implementing FCC and worked more directly with families had more positive perception towards family-centered practice in service delivery [11].

Despite the strong evidence-base and endorsement for FCC, however, research suggests that this framework has not been implemented globally and fully within services for children with disabilities [12]. Understanding which families experience FCC is important for addressing the remaining gaps; nevertheless, examining these gaps has been the focus of a few studies. In one study from Australia including parents of children aged 0–6 years with disabilities receiving early intervention services, families residing in cities, those whose children had early childhood teachers, and those who had an early childhood intervention professional coordinating the services, perceived receiving FCC to a greater extent [13]. Another study from Australia included families of children with physical disabilities from a rehabilitation center. Rural families with children younger than 6 years of age perceived more FCC and no associations were found between FCC and the level of parental education [14]. A study from the United States (US) described the perspectives of families of children with disabilities attending an outpatient pediatric rehabilitation facility. Receiving only one service versus multiple services was associated with higher levels of perceptions of FCC [15]. A study from Canada including parents of adolescents attending a neurology clinic found that higher parental mental scores were associated with FCC. The diagnoses of the adolescents, disease severity, parental marital status, age, ethnicity, and family income were not significantly associated with FCC [16].

Research is limited to only one quantitative and two qualitative studies on the family-centeredness of services for children with DS. In a quantitative study from the US, parental perceptions of family-provider relationships were examined in a sample of 110 mothers of children with DS. Mothers were generally satisfied with the family-centeredness of the care their children received and those receiving FCC reported feeling more satisfied and had higher levels of individual and family well-being [17]. A qualitative study from the US investigated the experiences of 37 service providers, and 13 parents of young children using focus groups and interviews. This study reported that communication between service providers and parents was inconsistent, uncoordinated or nonexistent [18]. Another qualitative study conducted in Australia investigated the experiences of nine families of children with DS and also identified gaps in the partnership between families and service providers [19]. 

Turkey provides an example of a middle-income country where accessible services for young children with disabilities do exist, but information on the family-centeredness of these services is lacking. Turkey has a population of 83 million and all children and youth which comprise 28% of the population are covered in the national health insurance. Since 2008, all children diagnosed with a disability receive government-subsidized services including special education, speech therapy, physical therapy and rehabilitation. Services that are received by young children are often similar to those for older children; two sessions per week, center-based, professional-led services. The term early intervention is not yet used in the legislation, and special education is the term used for services that address intellectual disabilities regardless of the age of the child. Special education and physical therapy are the most common type of services; occupational therapy and speech and language therapy are newly becoming available in large cities. Concepts of FCC are emerging, but still, children are typically separated from their caregivers for the duration of the intervention session and the professionals providing the interventions may have little contact with the families [20, 21]. Children with DS in Turkey are followed within the health system for primary care and for coexisting health problems. Since 2008, children with DS have been also eligible for government subsidized special services but whether these services comply with FCC principles have not been studied. Ankara University Developmental Pediatrics Division (AUDPD) is a center established in 2000 to provide services, training, research and advocacy for children with special needs. We aimed to determine to what degree children with DS, whom we followed and referred for services, received family-centered services and used a framework based on bioecological theory to examine child, family and service-related factors that were associated with the family-centeredness of the services. 

## 2. Material and methods 

### 2.1. Study design and sample 

We conducted a crosssectional observational study and recruited children with the diagnosis of Down syndrome who were followed at AUDPD between February 2020 and June 2020. Children were included if: a) they were accompanied by their mother for their visit to AUDPD; b) they were between the ages of 12–48 months; c) they had been attending at least one type of special service (special education, physical therapy and rehabilitation, occupational therapy or speech therapy) for at least 12 months; and d) the mother provided written consent for the study. The Ethics Committee of Ankara University School of Medicine approved the study.

### 2.2. Procedures

At the time of the AUDPD visit, one of the four developmental pediatricians provided a comprehensive developmental assessment based on principles of bioecological theory, the World Health Organization (WHO) International Classification of Functioning, Disability and Health (ICF), transdisciplinary, and FCC as well as a review of medical records and physical examination. Information related to the child such as whether the child had an accompanying illness was determined by the clinicians from the medical history, a comprehensive physical examination and consultations with other specialties or subspecialties. Health-related coexisting diagnoses were based on the International Classification of Diseases-10. Age appropriate standardized instruments including the International Guide for Monitoring Child Development [22, 23], Bayley scales of infant development version-III [24] or Vineland adaptive behavior scales-III [25] were used to assess the child’s development. The choice of the assessment tool was based on the child’s functioning and needs. Delay in development was defined as development below the equivalent of –2 standard deviations of the standardized mean on one or more of the domains of the instruments that were used. Information on psychosocial risk factors, and the type and the duration of the services was obtained through history taking during the assessment. At the end of the assessment, the clinicians applied the measure of process of care-20 (MPOC-20). 

### 2.3. Measures

The MPOC is a tool developed in Canada and is the most widely used tool globally in research to assess family-centered behaviors of service providers [26]. The original MPOC had 56 items that asked caregivers to rate their perceptions of the family-centeredness of the care they received from services on a seven-point Likert scale [27]. Subsequently, a 20-item version, the MPOC-20 was developed and its reliability and validity were established [28]. The MPOC-20 was selected for this study, as it has been shown to be reliable and valid in three middle-income countries, China [29], Jordan [30] and Brazil [31] and it allows for comparisons with prior research in high-income countries as well. 

MPOC-20 has 20 items, grouped into 5 subscales which represent essential aspects of family-centered services. The subscale enabling and partnership (EP) has 3 items; providing general information (PGI) has 5 items; providing specific information about the child (PSI) has 3 items; coordinated and comprehensive care for child and family (CCC) has 4 items; and respectful and supportive care (RSC) has 5 items. A higher score on the MPOC-20 corresponds with more favorable FCC. The seven-point Likert scale has the following ratings: 7 (to a very great extent), 6 (to a great extent), 5 (to a fairly great extent), 4 (to a moderate extent), 3 (to a small extent), 2 (to a very small extent), 1 (not at all), and with an additional “not applicable” category.

The Turkish version of the MPOC-20, certified and made available by the MPOC developers was purchased and applied in this study as instructed in the MPOC manual with one important modification. The reading level of the MPOC-20 is 8th grade, which is significantly higher than the 4th grade reading level of the majority of mothers served in our clinic. Problems in the applicability of the MPOC-20 for caregivers with low education have been previously reported from South Africa [32]. We therefore conducted a pilot study of 10 mothers and observed major difficulties in the self-administration of the MPOC-20. Most mothers were unable to sustain reading and comprehending the items. Only one mother was able to complete the tool without assistance from the researchers. Therefore, for this study, all the items of the MPOC-20 were read to all mothers by the clinicians assessing the child. As per standard instructions, mothers were asked, “to what extent do the people who work with your child do the following?” People working with the child were specified as the people who were providing services to the child in the center where the child was currently receiving services. As per the manual, the mother was asked to provide a general answer for all current service providers and services. If the mother had difficulty in understanding the items, explanations and examples were provided, and a visual 7-point Likert scale was shown. The developmental pediatricians did not make any comments about the services during the application of the MPOC-20. After the completion of the tool, however, if problems were identified in service delivery, these were discussed with the family and interventions were planned accordingly.

### 2.4. Data analysis

Descriptive statistics included frequencies for categorical data; means and standard deviations for normal continuous distributions; and medians and interquartile ranges otherwise. As instructed in the MPOC manual, we examined only subscale scores and did not use the total score of the MPOC-20. We first determined the strengths and weaknesses of the services by examining the subscale scores and distributions. We also determined which items were rated 7 (highest) and 1 (lowest) by over 50% of the mothers. The Shapiro–Wilk test was used to check whether there was a normal distribution of the numerical variables.

Based on bioecological theory we hypothesized that MPOC-20 scores would be associated with child, family and service-related factors. The Mann–Whitney U test was applied to determine whether there were statistically significant differences in the distributions of each of the five MPOC-20 subscale scores as continuous variables across the child, family and services related factors grouped as categorical variables. This was done because, the MPOC manual does not specifically advocate for a cut-off which should be used to categorize MPOC scores as optimal and most research has used subscale scores as continuous variables. The child related categorical variables examined were sex, age of child (≤ 24 months versus > 24 months), and chronic health conditions other than DS (present versus absent). Family related categorical variables included maternal education (< high school versus ≥ high school), income level (≤ minimum wage versus > minimum wage), psychosocial risk factors (one or more present versus absent). The services related factor examined was service duration (≤ 2 years versus > 2 years). There is one cut-off (≤ 4) on the MPOC-20 subscale scores that is referred to as below average in the MPOC-20 manual and prior research has used this to examine associated factors [33]. We therefore used this cut-off, so as to determine whether there were independent factors associated with MPOC-20 subscale scores. Using chi-square or Fisher’s exact tests where appropriate, we examined whether the categorical variables listed above were associated with MPOC-20 subscale scores using the ≤ 4 cut-off. Odd ratios (OR) and 95% confidence intervals (CIs) were computed. Finally, those factors found to be statistically significantly associated with MPOC-20 scores on the univariate analyses were entered a multivariate logistic regression model to determine independent factors associated with the dependent variable, MPOC-20 subscale scores ≤ 4. For statistical significance 95% confidence intervals (CIs) were used. Statistical analyses were conducted using IBM Statistical Package for Social Sciences 20.0 (IBM Corp., Armonk, NY, USA) package program.

## 3. Results

During the study period, 65 children with Down syndrome were eligible, and all mothers provided written consent to participate in the study. The sociodemographic characteristics of the children and families are shown in Table 1. Most children were boys (57%); their median age was 25.0 (IQR: 18.5–38.0) months. Most mothers (66.2%) and fathers (76.9%) had at least high school education. Most children (83%) were living in Ankara and receiving services in this city. All children were receiving special education, 79% were receiving physical therapy and 34% speech therapy. The median duration that the children had received the services that were evaluated with the MPOC-20 was 18.0 months (IQR: 12.0–27.0). 

**Table 1:** Sociodemographic characteristics of the sample.

	N	%
Boys	37	56.9
Child age (months)		
12–24	30	46.2
25–36	18	27.7
37–48	17	26.2
Maternal age (years)		
20–30	16	24.6
31–40	36	55.4
> 40	13	20.0
Paternal age (years)		
20–30	7	10.8
31–40	39	60.0
> 40	19	29.2
Maternal education		
Primary school or less	17	26.2
Secondary school	5	7.7
High school graduate	17	26.2
University education or higher	26	40.0
Paternal education		
Primary school or less	10	15.4
Secondary school	5	7.7
High school graduate	18	27.7
University education or higher	32	49.2
Family with multiple children	44	67.6
Family residing in Ankara	54	83.1

Table 2 shows the health and psychosocial characteristics of the children and families. A chronic health condition apart from Down syndrome was present in 71% of the children. All children had developmental delays in at least one domain of development. Most mothers (55%) reported at least one psychosocial risk factor: approximately one third expressed perceived stigma, a quarter expressed feelings of depression; and one fifth reported unemployment or financial difficulties.

**Table 2:** Chronic health conditions and psychosocial risk factors.

	N	%
Children with chronic health conditions		
Premature birth	24	36.9
Congenital heart disease	18	27.7
Hypothyroidism	12	18.5
Other conditions (gastrointestinal, hematological, immunological, neurological disorders, hearing impairment)	15	23.1
Psychosocial risk factors		
Perceived stigma	19	29.2
Mother’s feelings of depression	14	21.5
Unemployment, financial problems	11	16.9
Not getting enough support from friends and relatives	8	12.3
Father’s feelings of depression	7	10.8
Presence of illness in a family member	6	9.2
Marital problems	2	3.1

The results of the MPOC-20 subscale scores as continuous variables are shown in Table 3. The subscale score medians ranged from 3.0 (IQR: 4.38–6.50) to 6.0 (IQR: 4.80–6.80) and were highest for the subscales respectful and supportive care (RSC) and coordinated and comprehensive care for child and family (CCC). The subscales with the lowest median scores were providing specific information about the child (PSI) and providing general information (PGI). Items that were rated 7 (to a very great extent) by over 50% of the mothers were “look at the needs of your whole child”, and “provide opportunities for you to make decisions about treatment”. Items that were rated 1 (not at all) by over 50% of the mothers were “fully explain treatment choices to you” and “provide advice on how to get information or to contact other parents”.

**Table 3:** MPOC-20 subscale median scores and associated factors.

		Enabling and partnership	Providinggeneral information	Providing specific information	Coordinated and comprehensive care	Respectful and supportive care
	n (%)	Median (interquartile range)				
Total subscale	65 (100)	5.00 (4.00–6.00)	4.00 (3.00–5.00)	3.00 (4.38–6.50)	5.50 (4.38–6.50)	6.00 (4.80–6.80)
Sex						
Girls	28 (43.1)	4.83 (4.00–6.00)	4.20 (3.05–5.30)	3.00 (2.67–5.67)	5.25 (4.00–6.50)	5.90 (5.05–6.40)
Boys	37 (56.9)	5.33 (3.83–6.17)	3.80 (2.80–5.00)	3.33 (2.43–5.00)	5.75 (4.50–6.50)	6.20 (4.60–6.80)
Age						
≤ 24 months	30 (46.2)	4.67 (3.50–6.33)	4.00 (2.75–5.00)	3.17 (2.67–5.42)	5.63 (4.44–6.50)	5.60 (4.60–6.80)
> 24 months	35 (53.8)	5.33 (4.33–6.00)	4.20 (3.00–5.00)	3.00 (2.67–5.33)	5.25 (4.25–6.50)	6.20 (5.00–6.80)
Chronic conditions						
Present	46 (70.8)	5.00 (4.00–6.33)	4.40 (3.10–5.10)	3.17 (2.92–5.67)	5.63 (4.50–6.50)	6.10 (5.00–6.80)
Absent	19 (29.2)	5.33 (3.67–6.00)	3.60 (3.00–4.40)	3.00 (2.67–5.00)	5.25 (4.25–6.25)	5.40 (4.60–6.60)
Maternal education						
< high school	22 (33.8)	4.17 (2.67–5.42)*	3.80 (2.80–4.60)	3.33 (2.67–5.00)	4.75 (3.50–5.75)*	5.40 (3.75–6.60)*
≥ high school	43 (66.2)	5.67 (4.33–6.33)	4.20 (3.20–5.00)	3.00 (3.00–5.67)	6.00 (4.50–6.75)	6.20 (5.40–6.80)
Income						
≤ minimum wage	23 (35.4)	5.00 (4.00–6.33)	4.40 (2.80–5.00)	3.00 (2.67–5.00)	5.50 (3.50–6.50)	6.20 (4.00–6.60)
> minimum wage	42 (64.6)	5.00 (3.92–6.00)	3.80 (3.00–4.60)	3.17 (3.00–5.67)	5.50 (4.50–6.50)	5.80 (5.15–6.80)
Psychosocial risks						
Reported	36 (55.4)	5.00 (4.00–6.25)	3.90 (2.85–4.90)	3.00 (2.75–5.00)	5.63 (4.31–6.25)	6.30 (5.25–6.60)
Not reported	29 (44.6)	5.00 (4.00–6.00)	4.20 (3.10–5.40)	3.33 (2.67–5.67)	5.00 (4.38–6.75)	5.60 (4.30–6.90)
Service duration						
≤ 2 years	45 (69.2)	5.00 (4.00–6.08)	4.10 (3.00–4.70)	3.00 (2.67–5.08)	5.63 (4.50–6.50)	5.90 (4.90–6.80)
> 2 years	20 (30.8)	4.67 (3.67–6.00)	4.00 (2.80–5.20)	3.33 (2.67–5.67)	5.00 (4.00–6.50)	6.00 (4.60–6.60)
*P < 0.05, Mann–Whitney U test .						

The results of the univariate analyses using MPOC-20 scores as continuous variables and the child, family and service-related factors as categorical variables are also shown in Table 3. Mothers with education ≤ high school had statistically significantly lower scores on the following subscales: enabling and partnership (P = 0.014), coordinated and comprehensive care for child and family (P = 0.010) and respectful and supportive care (P = 0.030).

The results of the univariate categorical variable analyses are shown in Table 4. Mothers with education level < high school (OR = 6.75; 95%CI =1.77–25.64), and those with income ≤ minimum wage (OR = 3.94; 95%CI =1.10–14.02) were more likely to report RSC scores ≤ 4. Mothers with education level < high school (OR = 3.10; 95%CI = 1.06–9.05) were more likely to report EP scores ≤ 4. Multivariate logistic regression was performed only for the RSC subscale due to the two significant variables and showed that the variable that was independently associated with MPOC-20 scores was maternal education. When income was controlled for, mothers with education level < high school were more likely to have RSC scores ≤ 4 (OR = 5.13; 95%CI = 1.26–20.84) (Table 5).

**Table 4:** Bivariate analyses of factors associated with MPOC-20 subscale scores


		MPOC-20 subscale scores ≤ 4									
	Proportions	Enabling and partnership		Providinggeneral information		Providing specific information about the child		Coordinated and comprehensive care for the child and family		Respectful and supportive care	
	n (%)	n (%)	OR(95%CI)	n (%)	OR(95%CI)	n (%)	OR(95%CI)	n (%)	OR (95%CI)	n (%)	OR(95%CI)
Sex											
Girls	28 (43.1)	11 (39.3)	1.19 (0.43–3.29)	19 (67.9)	1.14(0.40–3.24)	18 (64.3)	0.86(0.30–2.43)	9 (32.1)	2.03(0.64–6.36)	5 (17.9)	0.78(0.22–2.73)
Boys	37 (56.9)	13 (35.1)		24 (64.9)		25 (67.6)		7 (18.9)		8(21.6)	
Age											
≤ 24 months	30 (46.2)	13 (43.3)	1.66(0.60–4.60)	19 (63.3)	0.79(0.28–2.21)	19 (63.3)	0.79(0.28–2.21)	7 (23.3)	0.87(0.28–2.73)	6 (20.0)	1.00(0.29–3.38)
> 24 months	35 (53.8)	11 (31.4)		24 (68.6)		24 (68.6)		9 (25.7)		7 (20.0)	
Chronic conditions											
Present	46 (70.8)	18 (39.1)	1.39(0.44–4.33)	28 (60.9)	0.41(0.11–1.45)	31 (67.4)	1.20(0.39–3.68)	11 (23.9)	0.88(0.25–2.99)	11 (23.9)	2.67(0.53–13.42)
Absent	19 (29.2)	6 (31.6)		15 (78.9)		12 (63.2)		5 (26.3)		2 (10.5)	
Maternal education											
< high school	22 (33.8)	12 (54.5)	3.10* (1.06–9.05)	16 (72.7)	1.58(0.51–4.86)	16 (72.7)	1.58(0.51–4.86)	7 (31.8)	1.76(0.55–5.62)	9 (40.9)	6.75*(1.77–25.64)
≥ high school	43 (66.2)	12 (27.9)		27 (62.8)		27 (62.8)		9 (20.9)		4 (9.3)	
Income											
≤ minimum wage	23 (35.4)	9 (39.1)	1.15(0.40–3.30)	12 (52.2)	0.38(0.13–1.12)	16 (69.6)	1.27(0.42–3.77)	7 (30.4)	1.60(0.50–5.08)	8 (34.8)	3.94*(1.10–14.02)
> minimum wage	42 (64.6)	15 (35.7)		31 (73.8)		27 (64.3)		9 (21.4)		5 (11.9)	
Psychosocial risks											
Reported	36 (55.4)	13 (36.1)	0.92(0.33–2.54)	24 (66.7)	1.05(0.37–2.95)	23 (63.9)	0.79(0.28–2.25)	9 (25.0)	1.04(0.33–3.26)	6 (16.7)	0.62(0.18–2.13
Not reported	29 (44.6)	11 (37.9)		19 (65.5)		20 (69.0)		7 (24.1)		7 (24.1)	
Duration of services											
≤ 2 years	45 (69.2)	15 (33.3)	0.61(0.20–1.79)	30 (66.7)	1.07(0.35–3.26)	30 (66.7)	1.07(0.35–3.26)	9 (20.0)	0.46(0.14–1.50)	8 (17.8)	0.64(0.18–2.30)
> 2 years	20 (30.8)	9 (45.0)		16 (65.0)		16 (65.0)		7 (35.0)		5 (25.0)	
* P < 0.05, chi-square test .											

**Table 5:** Multivariate logistic regression analyses of MPOC-20 respectful and supportive care subscale scores and associated factors.

	Respectful and supportive care subscale <4		
	OR	95% CI	P value
Maternal education < high school	5.13	1.26–20.84	0.02
Income < minimum wage	2.40	0.60–9.56	0.21

## 4. Discussion

This study has examined the family-centeredness of the services for children with Down syndrome and their families in a middle-income country. Mothers of children attending a developmental pediatrics clinic in Ankara, Turkey, reported on the family-centeredness of services using the MPOC-20. Most of the subscales median scores were at or above average; the subscale respectful and supportive care (RSC) had the highest whereas providing specific information about the child (PSI) had the lowest subscale scores. Using a bioecological theory-based model, among the child, family and service-related factors that were examined, the only variable that was found to be independently associated with MPOC-20 subscale scores was maternal education.

The MPOC has been the most widely used tool to assess family-centered behaviors of service providers globally [6,34] and has been used in studies on children with cerebral palsy [33], visual impairment [35], epilepsy [36], diabetes [37] and cancer [38]. As previous studies using the MPOC-20 specific to children with DS do not exist, we are able to make comparisons only with studies that include children with a variety of disabilities and health conditions. 

One of the main findings of our study relates to the specific subscale scores of the MPOC-20. Our study adds to the literature from numerous countries [6,34], Japan [8], Korea [9], China [10], Jordan [30] and South Africa [32] that have all reported that the RSC [6] or the EP [32] subscale scores rank highest and that the PGI or PSI subscale scores rank lowest. The evidence in the literature thus implies that service providers in both high and middle-income countries alike are better at providing respectful and supportive care but need to improve ways of providing information to families. Many countries differ with respect to culture, resources and risk factors related to childhood disability. Therefore, further research is still needed from different cultural and economic contexts on which components of FCC needs improvement and how these components can be improved efficiently.

Our second main finding is related to factors that are associated with MPOC-20 scores which we examined using a bioecological theory-based model. Based on prior literature and anecdotal observations we had hypothesized those children with DS who were boys [39], younger [14] or who have additional disorders or illnesses would need and receive more family-centered care and more information about the disorder. Child-related factors including sex, age, and whether the child had an accompanying disorder, however, were not associated with MPOC-20 scores. In our analyses, using MPOC-20 as continuous scores and with the recommended cut-off, maternal education was the factor associated with subscale scores related to respectful and supportive care. There may be three explanations for this important finding: a) mothers with higher education may have skills in demanding and eliciting better FCC from service providers; b) service providers may be more likely to partner with higher educated mothers in providing FCC; c) mothers with higher education may report on the services they receive more favorably that those with lower education even when the family-centeredness of services are the same for both groups. The fact that maternal education was not significantly associated with all subscales and that provision of information scored low even for mothers with higher education suggests that service providers may be better at engaging with mothers with higher education but may provide inadequate information regardless of mothers’ educational level. It will be important for future research to determine efficacious ways of equalizing respectful and supportive care for families with all educational backgrounds while at the same time improving on provision of information for all families. We had also hypothesized that other family-related factors such as presence of psychosocial stressors and service duration would be associated with greater FCC, but this also was not demonstrated in our study. Due to the differences in the samples, methodologies and the factors examined, it is not possible to draw generalizable information from prior studies on which factors are associated with family-centeredness of services. For example, even in the two studies from Australia, the results were contradicting such that in one study urban [13] and in the other rural [14] place of residence were associated with higher family-centeredness. Multiple studies have indicated that those children who had a key person who was coordinating services [13, 39] or who attended single versus multiple services [15] had higher MPOC scores. We were not able to determine the influence of this key factor because in our sample, all children had a care coordinator who was the AUDPD clinician, but this was not done in any of the community services. 

The high rate of recruitment is an important strength of this study; this enabled all children within a time frame to be included and selection bias to be avoided. The pilot study we conducted was a further strength and provided us with information related to how we would apply the MPOC. The researchers applied the more cumbersome and time-consuming technique to increase the understanding of mothers and reduce missing data. The homogeneity of our age range enables more specific information on services reaching young children. The single urban center setting which limits generalizability is the main limitation of our study. We were unable to analyze differences between children living in urban versus rural regions because although some referrals came from outside of the city, we did not have a large enough sample size for children who received services outside of Ankara and in rural areas. Future studies are needed other countries to provide more generalizable information on the family-centeredness of services globally. 

## 5. Conclusion

Down syndrome exemplifies a well-known disability that benefits greatly from family-centered early intervention services and Turkey exemplifies a middle-income country where government subsidized services do exist for all children with disabilities. Our findings imply that there are strengths and limitations in the family-centeredness of services received by children with DS. Whereas respectful care is often provided, there are important deficiencies in the provision of information that is greatly needed by families. Our findings imply that interventions to improve FCC, particularly for families with lower education are greatly needed in Turkey and likely other countries. The reasons for the discrepancies in the family-centeredness of services for mothers with different educational levels need to be urgently determined and addressed.

## Financial disclosure

The authors declare no financial disclosures. 

## Source of support

There is no source of support. 

## Informed consent

The study protocol received institutional review board approval and that all participants provided informed consent in the format required by the relevant authorities and/or boards. Ankara University School of Medicine Ethics Committee approved study (Approval number: 1219226, 13 January 2020).
